# CT-derived measures of muscle quantity and quality predict poorer outcomes from elective colorectal surgery: a UK multicentre retrospective cohort study

**DOI:** 10.1007/s10151-023-02769-3

**Published:** 2023-05-03

**Authors:** J. E. M. Blackwell, P. J. J. Herrod, B. Doleman, H. Boyd-Carson, D. Dolan, L. Wheldon, S. R. Brown, A. Banerjea, S. Moug, J. N. Lund, The POMPOMM Collaborative, Michael Wong, Alexander N. C. Boucher, Ross Sian, Peter Metherall, Jenna Harthorne, Natasha Redhead, Leonie Walker, Fiona Marshall, Christopher G. D. Clarke, Paul Thomas, Liam Hyland, Jacob R. Hatt, Edward Hardy, Thomas Smart, James Bunce, Alysha Careless, Yi Lin Lai, Elizabeth Gemmill

**Affiliations:** 1 Royal Derby Hospital, Derby, England UK; 2 Queens Medical Centre, Nottingham, England UK; 3 King’s Mill Hospital, Nottinghamshire, England UK; 4 The Northern General Hospital, Sheffield, England UK; 5 Royal Alexandra Hospital, Paisley, Scotland UK; 6 University of Nottingham, Nottingham, England UK; 7 University of Glasgow, Glasgow, Scotland UK; 8 University of Sheffield, Sheffield, England UK

**Keywords:** Psoas muscle, Sarcopenia, Myosteatosis, Lean muscle

## Abstract

**Purpose:**

To assess whether preoperative radiologically defined lean muscle measures are associated with adverse clinical outcomes in patients undergoing elective surgery for colorectal cancer.

**Methods:**

This retrospective UK-based multicentre data collection study identified patients having had colorectal cancer resection with curative intent between January 2013 to December 2016. Preoperative computed-tomography (CT) scans were used to measure psoas muscle characteristics. Clinical records provided postoperative morbidity and mortality data.

**Results:**

This study included 1122 patients. The cohort was separated into a combined group (patients with both sarcopenia and myosteatosis) and others group (either sarcopenia or myosteatosis, or neither). For the combined group, anastomotic leak was predicted on univariate (OR 4.1, 95% CI 1.43–11.79; *p* = 0.009) and multivariate analysis (OR 4.37, 95% CI 1.41–13.53; *p* = 0.01). Also for the combined group, mortality (up to 5 years postoperatively) was predicted on univariate (HR 2.41, 95% CI 1.64–3.52; *p* < 0.001) and multivariate analysis (HR 1.93, 95% CI 1.28–2.89; *p* = 0.002). A strong correlation exists between freehand-drawn region of interest-derived psoas density measurement and using the ellipse tool (*R*^2^ = 81%; *p* < 0.001).

**Conclusion:**

Measures of lean muscle quality and quantity, which predict important clinical outcomes, can be quickly and easily taken from routine preoperative imaging in patients being considered for colorectal cancer surgery. As poor muscle mass and quality are again shown to predict poorer clinical outcomes, these should be proactively targeted within prehabilitation, perioperative and rehabilitation phases to minimise negative impact of these pathological states.

## Introduction

Predicting complications following major surgery is difficult in frail elderly populations undergoing colorectal cancer resection. Risk prediction models suffer methodological limitations and their utility in elderly cohorts, most at risk of complications, is unclear [1, 2]. Objective physical measures such as the Timed Up and Go test and hand grip strength correlate with physical fitness levels in the general population; however, standardised cut-offs are yet to be defined within specific comorbid groups matching those undergoing colorectal surgery [3]. The gold standard assessment of cardiorespiratory fitness, the cardiopulmonary exercise test (CPET), is not universally available within the UK [4]. There remains a need for objectively measured markers of individual patient status to more accurately stratify individual risk associated with major surgical resection.

Quantification of lean muscle mass (and associated disease states of sarcopenia and myosteatosis) has been proposed as a metric for individualised risk stratification and outcome prediction for patients with colorectal cancer undergoing surgery [5–7]. Sarcopenia, the combined loss of muscle mass and function with age, is a significant predictor of major complications following abdominal surgery and is associated with adverse oncological outcomes [8, 9]. Myosteatosis, fatty infiltration into muscles, is associated with diabetes and obesity, reduced muscle activity, myositis and cancer [10]. Myosteatosis increases hospital length of stay and readmissions following cancer surgery [11]. Sarcopenia and myosteatosis have independent negative effects on overall survival in patients with colorectal cancer [9, 12–14]. The literature suggests the joint effect of sarcopenia and myosteatosis is additive (rather than multiplicative) and highly predictive of reduced overall, recurrence-free, and cancer-specific survival compared with normal patients [12, 14].

Preoperative identification of radiologically defined pathological states has shown promising predictive values and propensity toward surgical complications and reduced overall survival [5, 8, 15]. Objective muscle measurements from computed tomography (CT) scans are particularly appealing as this is the standard preoperative radiological investigation in the UK when investigating and staging colorectal cancer. Cross-sectional area of the psoas muscle at the third lumbar vertebral level (L3) on preoperative CT scans can be used to assess whole body lean muscle mass, and when normalised for height is used as a quantitative index of sarcopenia (referred to in this paper as psoas muscle index (PMI) (synonymous with total psoas index (TPI) [15]) [5, 8, 15]. A CT-derived measure of lean muscle quality is psoas muscle density (PD) (or muscle radio attenuation) and is measured in Hounsfield units (HU), with lower PD values indicating myosteatosis [11]. Measurements of both cross-sectional area and density can be quickly attained with limited training, without the requirement of specialist software [5].

Conflicting opinion exists in the literature regarding definitions, exact measurement and clinical utility of these radiologically derived values [5, 6], particularly as normal reference ranges for age, gender and comorbidities are yet to be fully defined [13]. Developing our understanding of variation in measurement techniques could further improve efficacy of measurement and accessibility of this measurement for incorporation within clinical settings.

Identifying patients at high risk of complications, allowing for optimal advance resource allocation (such as high dependency beds) through ubiquitous, quick and easily measured parameters is clinically appealing. Understanding an individual’s specific risk facilitates a more meaningful informed consent process and should enhance shared decision-making when considering postoperative quality of life and certain adjuncts to primary resection (i.e. diverting or defunctioning stoma), which can minimise the impact of anastomotic leak should it occur [16]. Individualised risk assessment, in the fully informed patient, may even provide evidence for the decision to avoid anastomosis entirely.

## Aims

This paper aims to investigate whether the use of preoperative radiologically defined lean muscle measures is associated with adverse clinical outcomes in patients undergoing elective surgery for colorectal cancer.

## Methods

### Ethics

This study was granted NHS ethics approval (IRAS 273242) with local governance procedures followed at separate sites.

### Patient group

This retrospective UK-based multicentre data collection study identified patients having had colorectal cancer resection with curative intent between January 2013 and December 2016. Centres included were Royal Derby Hospital (Derby, England), Queens Medical Centre (Nottingham, England), King’s Mill Hospital (Nottinghamshire, England), The Northern General Hospital (Sheffield, England) and Royal Alexandra Hospital (Paisely, Scotland). Patient cohorts were identified from individual hospital cancer records and cross-referenced with the National Bowel Cancer Audit data. Encrypted databases were kept at each site with central study members conducting data analysis blinded to patient site. Site leads were responsible for maintaining validity of locally collected data. Data extractors received written instructions with verbal troubleshooting from the central research team (due to COVID travel restrictions preventing in-person visits).

### Computed tomography image data extraction

**Fig. 1:**
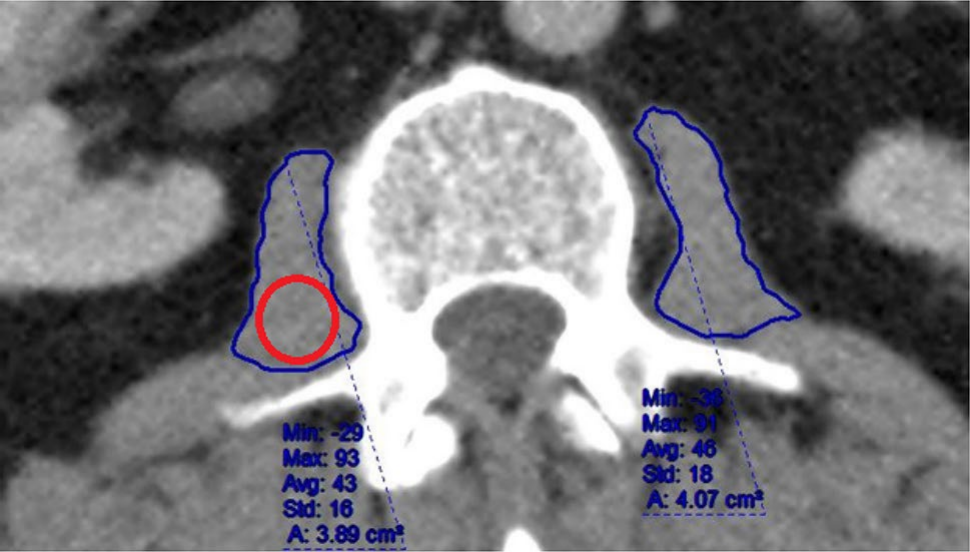
Psoas muscle measurement. Freehand region of interest (ROI) drawn bilaterally (blue). Example of ellipse tool use for ROI on right psoas muscle body (red)

All patients had preoperative contrast-enhanced portal-venous CT scans. Psoas muscle analysis used the most superior CT slice in which both transverse processes of the third lumbar vertebrae (L3) were visible as previously described [5, 17] (Fig. 1) using the hospital standard PACS imaging software (Centricity Universal Viewer Version 6.0, GE Healthcare, Chicago, USA) with one institution using Mimcloud (MIMsoftware inc., Cleveland, USA). Freehand regions of interest (ROI) were drawn around both psoas muscles in the same CT slice, excluding macroscopically obvious fat infiltration. The automatically calculated cross-sectional area and mean density in HU of each ROI was recorded. The ‘ellipse’ tool was used to draw the largest possible ellipse-shaped ROI within the psoas muscle borders in the same CT slice, recording the mean density in HU of this ellipse. The arithmetic mean of the density of both psoas muscles and both ellipses was then calculated. The PMI was calculated as combined left and right psoas muscle cross-sectional area normalised by the patient’s height [(right CSA + left CSA)/height^2^(cm^2^/m^2^)] [18].

### Clinical outcome data

Electronic patient records provided pre- and postoperative clinical details. The Clavien–Dindo (CD) system was used to classify postoperative complications with CD 3/4 complications defined as major complications [19]. Anastomotic leak was defined as proven leaks requiring intervention (i.e. CD 3/4). Intervention due to anastomotic leak was cross-referenced with theatre and interventional radiology records to ensure complete data capture. Postoperative complication data was collected until discharge from index operation (with electronic records checked for readmissions up to 30 days) and mortality data was captured for 5 years postoperatively.

### Statistical analysis

Variables included mean psoas density (PD, measured in HU) and PMI. We calculated PMI as the sum of psoas muscle areas (right and left) divided by height squared. We defined myosteatosis as those with PMI < 41 HU if body mass index (BMI) < 25 and PMI < 33 HU if BMI > 25 [9]. We defined sarcopenia as a PMI < 5.86 cm^2^/m^2^ for men and MPI < 4.56 cm^2^/m^2^ for women [7]. We also identified patients with both sarcopenia and myosteatosis to assess whether clinical outcomes were poorer within this group.

Descriptive data of baseline characteristics are presented as median [interquartile range] or number (%) as appropriate. Differences were tested using standardised differences with > 0.1 taken to mean baseline imbalance. To test the association between sarcopenia/myosteatosis and binary outcomes we performed logistic regression. For anastomotic leak, we excluded patients who had a stoma. Estimates were adjusted for a priori confounding variables including age at diagnosis (< 60 years, 60–80 years and over 80 years old), sex, chronic kidney disease (CKD) stage 4 and 5, severe anaemia (Hb < 100 g/L), BMI (underweight, normal/overweight and obesity) and hypoalbuminaemia (albumin < 35 g/L). We assessed continuous variables for linearity using plots of the variable versus logit transformed outcomes.

We did not impute missing data, and missing data were case-wise deleted from multivariate models. As a result of issues with perfect separation, we used Firth logistic models with penalised maximum likelihood estimates where appropriate. Time to event data was analysed using Cox proportional hazards models. Length of stay was analysed using negative binomial regression. Binary outcomes are presented as odds ratios (OR), time to event data as hazard ratios (HR) and length of stay as incident rate ratios (IRR). We also assessed whether mean ellipse could predict mean psoas density using linear regression. We conducted a multivariate sensitivity analysis evaluating mortality at 1 year to identify if a shorter follow-up period changed estimates. All estimates are presented with 95% confidence intervals (CI) and* p* values. All analyses were performed using Stata Version 16.

## Results

We included 1122 patients. Missing data meant that BMI could not be calculated in 114 patients (10%) and height for 116 patients (10%). As group definitions required these variables in their calculation, numbers were reduced accordingly. Other missing data included albumin (8%) and length of stay (2%). All other variables had less than 1% missing data. Anthropometric and baseline clinical data can be seen in Table 1. For the purposes of this paper the ‘Combined’ group represents those patients with both radiological sarcopenia and myosteatosis; the group named ‘Others’ contains patients who have either radiologically normal muscle measures or sarcopenia/myosteatosis alone.

**Table 1:** Anthropometric and baseline data

	Total (*n* = 1122)	Combined group (*n* = 82)	Others (*n* = 919)	Standardised difference
Age	70 [61–76]	75 [69–81]	69 [61–75]	0.72
Sex (F)	473/1119 (42)	20/82 (24)	396/917 (43)	0.41
BMI (kg/m^2^)	27.2 [23.9–30.4]	24.5 [22.5–28.4]	27.5 [24.1–30.5]	0.30
eGFR (ml/min)	63 [60–87]	60 [58–70]	60 [60–86]	0.42
Haemoglobin (g/L)	129 [114–142]	120 [112–133]	130 [114–142]	0.27
Albumin	38 [34–41]	35 [32–37]	38 [35–41]	0.76

Complication event rates (*n* = 1122)	
Anastomotic leak	3.6%
CD 3–5 complications	8.4%
Mortality during follow-up	24.5%

### Predicting psoas density from ellipse

**Fig. 2:**
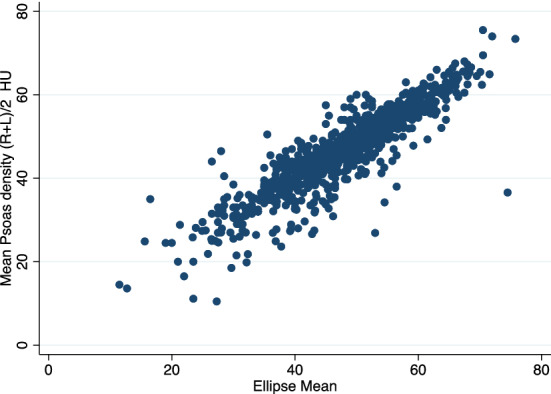
Graph showing the correlation between freehand-drawn mean psoas density (HU) and single ellipse (HU) used to capture psoas density on the same slice at L3

Mean ellipse was a significant predictor of psoas density (*R*^2^ = 81%; *p* < 0.001) (Fig. 2). Mean ellipse did not predict mean CSA (*R*^2^ = 0%; *p* = 0.98).

### Major complications

Myosteatosis did not predict major complications on univariate (OR 1.48, 95% CI 0.78–2.84; *p* = 0.23) or multivariate analysis (OR 0.97, 95% CI 0.47–1.99; *p* = 0.93). Sarcopenia did not predict major complications on univariate (OR 1.51, 95% CI 0.9–2.53; *p* = 0.12) or multivariate analysis (OR 1.19, 95% CI 0.67–2.1; *p* = 0.55). For the combined group, major complications were almost predicted on univariate (OR 1.9, 95% CI 0.96–3.76; *p* = 0.06) but not multivariate analysis (OR 1.1, 95% CI 0.5–2.4; *p* = 0.81).

### Anastomotic leak

There were 702 participants who did not have a defunctioning or diverting stoma who were included in this outcome. Myosteatosis predicted anastomotic leak on univariate (OR 2.82, 95% CI 1.00–8.00; *p* = 0.05) and multivariate analysis (OR 3.13, 95% CI 1.05–9.33; *p* = 0.04). Sarcopenia predicted anastomotic leak on univariate (OR 4.97, 95% CI 1.14–21.6; *p* = 0.03) and multivariate analysis (OR 3.97, 95% CI 1.01–15.61; *p* = 0.05). For the combined group, anastomotic leak was predicted on univariate (OR 4.1, 95% CI 1.43–11.79; *p* = 0.009) and multivariate analysis (OR 4.37, 95% CI 1.41–13.53; *p* = 0.01).

### Length of stay

Myosteatosis predicted increases in length of stay on univariate (IRR 1.21, 95% CI 1.06–1.38; *p* = 0.004) but not on multivariate analysis (IRR 1.10, 95% CI 0.96–1.25; *p* = 0.16). Sarcopenia predicted length of stay on univariate (IRR 1.11, 95% CI 1.02–1.21; *p* = 0.02) and multivariate analysis (IRR 1.1, 95% CI 1–1.2; *p* = 0.04). For the combined group, length of stay was predicted on univariate (IRR 1.27, 95% CI 1.1–1.47; *p* = 0.001) but not on multivariate analysis (IRR 1.14, 95% CI 0.98–1.32; *p* = 0.09).

### Mortality

**Fig. 3:**
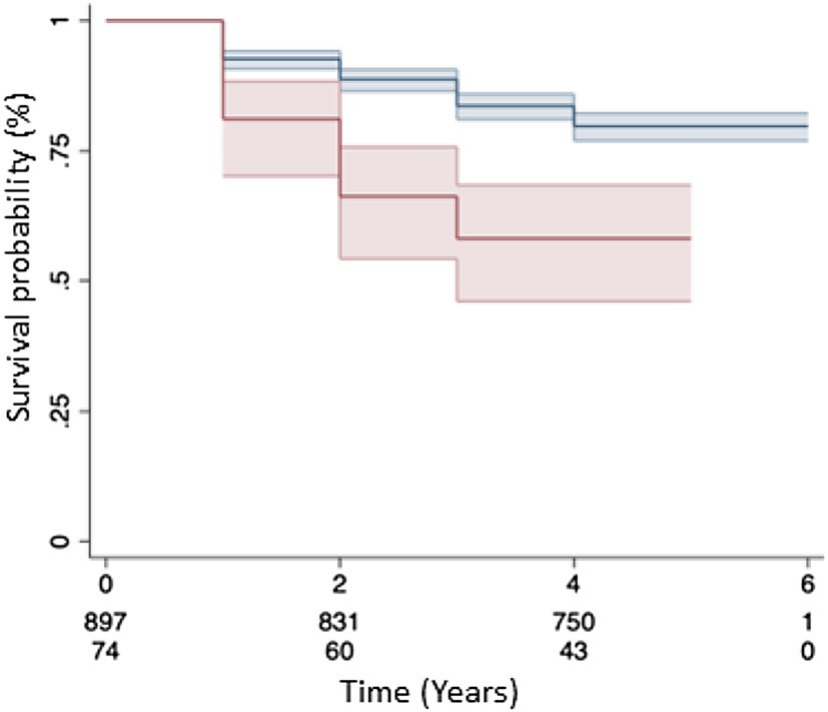
Kaplan–Meier chart showing mortality over time. Red line = Combined group (both sarcopenia and myosteatosis), Blue line = Others (rest of patients in study), Red/Blue shaded areas 95% confidence intervals

Myosteatosis predicted increases in mortality on univariate (HR 1.89, 95% CI 1.31–2.71; *p* = 0.001) and multivariate analysis (HR 1.61, 95% CI 1.09–2.36; *p* = 0.02). Sarcopenia did not predict mortality on univariate (HR 1.3, 95% CI 0.97–1.75; *p* = 0.08) or multivariate analysis (HR 1.12, 95% CI 0.82–1.54; *p* = 0.49). For the combined group, mortality was predicted on univariate (HR 2.41, 95% CI 1.64–3.52; *p* < 0.001) and multivariate analysis (HR 1.93, 95% CI 1.28–2.89; *p* = 0.002). Figure 3 shows mortality over time comparing the combined group versus the rest of the study patients.

On multivariate sensitivity analysis of mortality at 1 year, myosteatosis predicted mortality (OR 2.01, 95% CI 1.17–3.48; *p* = 0.01). As with the main analysis, sarcopenia did not predict mortality at 1 year although estimates were imprecise (OR 1.55, 95% CI 0.93–2.59; *p* = 0.09). For the combined group, mortality was predicted on multivariate analysis (OR 2.35, 95% CI 1.31–4.22; *p* = 0.004).

## Discussion

This multicentre study adds the largest UK patient cohort to the growing literature utilising radiologically defined lean muscle parameters (psoas density and myosteatosis) to predict clinical outcomes in patients undergoing elective colorectal cancer surgery. Myosteatosis may have a greater clinical utility as a simple, easily measured, reproducible and ubiquitous parameter than CT-derived sarcopenia in this group. Patients presenting with both myosteatosis and sarcopenia appears to represent a higher-risk group of patients as shown previously [12, 14]. Additionally, this study shows that the density within a simple ellipse ROI drawn within the psoas muscle using a standard freehand tool (requiring no training or specialist image analysis software) correlates well to previously described methods of obtaining density from freehand-drawn ROIs around psoas muscles. These measures can complement other variables currently used to predict individual surgical risk. They should be incorporated routinely in staging CT scan reports for discussion at the colorectal multidisciplinary team meeting (MDT) when assessing functional status and suitability for surgery.

Myosteatosis and sarcopenia both independently predicted anastomotic leak, in keeping with previous literature [5]. Our group also however previously demonstrated that low psoas density predicted Clavien–Dindo grade 3 or 4 complications in a smaller cohort (1.65–24.23) *p* = 0.007, adjusted OR 14.37 (1.37–150.04) *p* = 0.026 [5], which was not apparent in this much larger cohort. This discrepancy may be explained by the small, single-centre nature of the previous study with a much lower event rate and also by the multifactorial causative nature of postoperative complications, which will have reduced precision as previously acknowledged [5, 20]. We saw a trend toward the group of patients with both sarcopenia and myosteatosis having significantly more serious postoperative complications. With further investigation and a larger sample size this relationship may prove to be more apparent as previously published studies from other centres highlighting the group of patients suffering both myosteatosis and sarcopenia [12, 14], who may be a clinically important cohort to consider when assessing fitness for surgery and discussing individualised relative risks.

Both sarcopenia and myosteatosis were associated with increased length of stay as seen previously [15, 21]. However, the factors determining postoperative length of stay are numerous of which postoperative surgical complications are only one component. It has been previously shown that sarcopenia in older adults is a risk factor for the requirement for future care home use [22] and is associated with an increased length of stay in medical as well as surgical patients [23]. As a result of the retrospective nature of this study, social circumstances and the additional time spent in hospital for other reasons (i.e. stoma training, physiotherapy/occupational therapy requirements) were not assessed which may have reduced the accuracy of our estimation of the effects of muscle measures on length of stay.

Patients with myosteatosis had a significant increase in the risk of death in the 5 years following resection compared with those without myosteatosis. As expected, patients with both reduced muscle quality and quantity appeared to have a twice the risk of death (up to approx. 5 years) and is in keeping with a previous systematic review of the impact radiologically determined sarcopenia on survival after all types of abdominal surgery [8]. Myosteatosis may reflect global frailty and malnutrition at presentation, leading to reduced muscle quality, which we know carries a poorer prognosis following major surgery [2, 24]. Sarcopenia alone did not predict mortality within this study. This contrasts with previous studies having shown higher short-term (30 days and 1 year) [15] and longer-term mortality rates in sarcopenic patients with colorectal cancer [9, 12]. It may also reflect of the omission of muscle function when measuring radiological sarcopenia (from the true definition of sarcopenia) [25]. Psoas muscle density may more accurately represent general deconditioning and poor skeletal muscle quality (as judged by presence of myosteatosis) versus radiologically defined sarcopenia. This study did not measure muscle function, which could explain why we saw a lower than expected rate of complications/mortality in the sarcopenic group (as there may have been radiologically sarcopenic patients with preserved muscle function [25]). We know that preserved muscle function aides early postoperative mobilisation and reduces complications and length of stay following colorectal surgery [26], which is unsurprising given that simple tasks such as sit to stand require optimal functioning of the trunk muscles (including the psoas muscle). The above uncertainties highlight the need for larger-scale clinical population-based studies to define normal cut-offs in patient groups. Interpretation of muscle quantity, quality and function, both independently and combined, is of paramount importance when aiming to individualise preoperative risk assessment. This may allow muscle quality/quantity and concurrent function to be targeted by prehabilitation and perioperative strategies which could lead to improvements in clinical outcomes [20, 27, 28].

Poor muscle quantity and quality represent chronic deconditioning of skeletal lean muscle mass and are associated with poorer short- and long-term outcomes [8, 13]. Understanding the true meaning of these measures is important (as discussed above), but more so is the consideration of their clinical application to help identify and reduce overall surgical risk to the patient. Reduced values are often multifactorial secondary to chronic health conditions (i.e. anaemia, diabetes, cardiorespiratory disease, malnutrition) as well as the underlying disease process of concern (i.e. cancer) and should be pragmatically interpreted as such. Radiologically defined parameters of sarcopenia and myosteatosis can aid preoperative nutritional assessment by adding detail to individualised patient assessment versus traditional methods (i.e. BMI, weight loss) [11] and have been incorporated within prognostic models for patients diagnosed with colorectal cancer [7]. Psoas density measurement has been proposed to aid real-time decision-making in the colorectal MDT and could add to the individualised counselling of a patient’s specific risk of complication and death from major surgical resection [5].

### Limitations

This paper used previously published cut-off values for myosteatosis and sarcopenia; however, further large-scale cohort studies are required to clearly define these cut-off values in both health and disease states in addition to cancer groups [9, 13]. Variation in methodological approach for obtaining body composition measures has been acknowledged within the literature previously [9, 13]. These factors limit the generalizability of our findings. It is also acknowledged that radiological assessment of sarcopenia alone is probably insufficient to diagnose true sarcopenia without concomitant evidence of decreased muscle function [25]. Preoperative assessment should include radiological muscle quality/quantity assessment as well as functional assessment of muscle strength and performance. This study was a retrospective data collection study and has inherent limitations as such. We attempted to mitigate convenience sampling by recording consecutive cases meeting the inclusion criteria through each unit over the study time period. We accept the risk of misclassification bias given the study period was longer than 5 years previously. We cannot account for any complication beyond discharge wherein the patient presented to a different hospital to that where they had their primary cancer resection.

## Conclusion

Measures of lean muscle quality and quantity, which predict important clinical outcomes, can be quickly and easily taken from routine preoperative imaging in patients being considered for colorectal cancer surgery. As poor muscle mass and quality are again shown to predict poorer clinical outcomes, these should be proactively targeted within prehabilitation, perioperative and rehabilitation phases to minimise negative impact of these pathological states. Further work should include incorporation of muscle measures in prospective cohorts for preoperative risk assessment and explore its efficacy as an objective measure for enhancing the informed consent process.

## Data Availability

The datasets generated during and/or analysed during the current study are available from the corresponding author on reasonable request.

## Appendix

See Tables 2 and 3.

**Table 2:** Breakdown of operations performed in patient cohort

Operations performed	Total	Female	Male
	*n* = 1122 (%)	*n* = 477 (%)	*n* = 645 (%)
Right sided (right hemicolectomy, extended right hemicolectomy)	402 (39)	178 (44)	224 (56)
Left sided (anterior resection, sigmoid colectomy, left colectomy, Hartmanns, abdomino-perineal resection of rectum, extra-levator excision of rectum)	679 (61)	281(41)	398 (59)
Total/subtotal colectomy	41 (4)	18 (44)	23 (56)

**Table 3:** Univariate and multivariate predictors for the primary outcome of anastomotic leak (if no stoma) and combined group

Variable	Univariate analysis	Multivariate analysis
Combined group	4.1 (1.43–11.79)*	4.37 (1.41–13.53)*
Age group (< 60 years reference)		
60–80 years	1.78 (0.52–6.1)	0.91 (0.27–3.08)
80+ years	0.74 (0.12–4.52)	0.24 (0.03–1.89)
Sex (female reference)	1.88 (0.8–4.42)	1.98 (0.73–5.43)
CKD 4 and 5	2.92 (0.15–55.73)	3.62 (0.18–72.58)
Anaemia	1.01 (0.3–3.47)	1.73 (0.48–6.19)
BMI (normal reference)		
Obesity	0.91 (0.32–2.56)	0.75 (0.26–2.2)
Underweight	2.59 (0.31–21.32)	3.44 (0.51–23)
Low albumin	1.02 (0.4–2.64)	1.07 (0.36–3.12)

Estimates presented as odds ratios (95% confidence intervals)

*Denotes statistical significance
